# Identification of antibiotics for use in selection of the chytrid fungi *Batrachochytrium dendrobatidis* and *Batrachochytrium salamandrivorans*

**DOI:** 10.1371/journal.pone.0240480

**Published:** 2020-10-20

**Authors:** Kristyn A. Robinson, Mallory Dunn, Shane P. Hussey, Lillian K. Fritz-Laylin

**Affiliations:** Department of Biology, The University of Massachusetts Amherst, Amherst, MA, United States of America; Vanderbilt University School of Medicine, UNITED STATES

## Abstract

Global amphibian populations are being decimated by chytridiomycosis, a deadly skin infection caused by the fungal pathogens *Batrachochytrium dendrobatidis* (*Bd*) and *B*. *salamandrivorans* (*Bsal*). Although ongoing efforts are attempting to limit the spread of these infections, targeted treatments are necessary to manage the disease. Currently, no tools for genetic manipulation are available to identify and test specific drug targets in these fungi. To facilitate the development of genetic tools in *Bd* and *Bsal*, we have tested five commonly used antibiotics with available resistance genes: Hygromycin, Blasticidin, Puromycin, Zeocin, and Neomycin. We have identified effective concentrations of each for selection in both liquid culture and on solid media. These concentrations are within the range of concentrations used for selecting genetically modified cells from a variety of other eukaryotic species.

## Introduction

**Fig 1:**
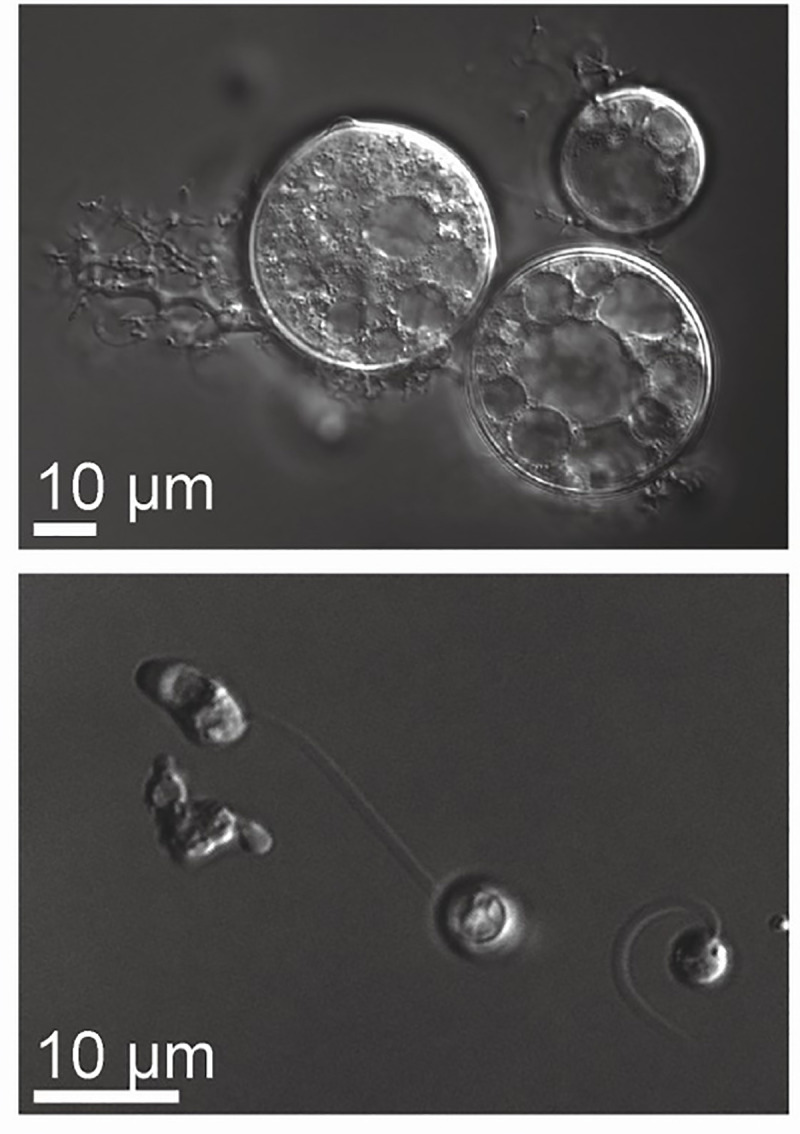
Life cycle of chytrid fungi. As illustrated here with images of *Bsal*, chytrid fungi have a biphasic life cycle characterized by a stationary growth phase called a sporangium (top) and a motile dispersal phase called a zoospore (bottom). Images taken at 100X using differential interference contrast (DIC) microscopy.

Chytrids are early diverging fungi that are commonly found in aquatic and moist environments [1]. They play key ecological roles, particularly by cycling carbon between trophic levels [2, 3]. Chytrids have a biphasic life cycle characterized by motile and sessile stages (**Fig 1**) [4–6]. They begin their life as motile “zoospores,” which use a flagellum to swim through water and, for some species, actin-based motility to crawl along surfaces [7, 8]. Zoospores then transition to a sessile growth stage by retracting their flagellum and building a cell wall in a process referred to as encystation. Encysted spores of many species develop into sporangia and develop hyphal-like structures called rhizoids and grow rapidly. Each sporangium produces many zoospores that exit via discharge papillae to begin the life cycle anew.

Many chytrids are pathogens that infect protists, plants, algae, fungi, and vertebrates [2]. The most infamous chytrids are the vertebrate pathogens *Batrachochytrium dendrobatidis* (*Bd*) and *B*. *salamandrivorans* (*Bsal*). Both pathogens cause chytridiomycosis, a skin disease plaguing amphibians worldwide [4, 6]. Recent estimates indicate that *Bd* has affected several hundred amphibian species and has been recorded on every continent except for Antarctica [9–11]. *Bsal* was more recently discovered in 2013 after a steep decline in fire salamander populations in Belgium [6].

Management strategies for these pathogens have been developed and implemented in limited contexts, but implementation in real world settings remains a challenge. To develop better treatments, we need to understand the biology of chytrids in order to identify targets for drug development. However, studying the molecular mechanisms driving pathogenesis remains challenging due to the lack of genetic tools available for chytrid fungi. Electroporation protocols have been developed for *Bd* and *Bsal*, which could be used to deliver molecular payloads for genetics manipulation such as plasmids and/or CRISPR-Cas9 complexes [12]. The recent success in genetic manipulation of a related chytrid species, *Spizellomyces punctatus* (*Sp*), is a major breakthrough for our ability to study chytrid biology [8]. We and others are now striving to adapt this technology to *Bd* and *Bsal* to further our understanding of chytridiomycosis.

A key step to genetic tool development is the identification of methods for selection of successful transformants. The most commonly used selection method is antibiotic resistance: incorporating a gene that provides specific drug resistance allows transformed cells to survive exposure to the antibiotic while all of the other cells are killed [13]. Distinct classes of antibiotics are commonly used for selection, each with their own molecular targets and corresponding organismal specificity. In addition to testing whether a given antibiotic kills cells of interest, it is important to pay attention to the effective concentration of each antibiotic. This is because a low concentration will not apply sufficient selective pressure and a high concentration could produce off-target effects and kill cells indiscriminately [14].

In this paper, we examine five antibiotics used in fungal and animal systems and identify the effective inhibitory concentration(s) necessary to prevent cell growth in liquid and solid media. Hygromycin, Blasticidin, and Puromycin inhibit protein translation in both bacterial and eukaryotic cells. Hygromycin inhibits protein synthesis by binding to the small ribosomal subunit and stabilizing the tRNA in the A site, preventing the progression of translation [15]. Blasticidin inhibits the terminating step of translation while Puromycin causes the ribosome to prematurely detach from mRNA [16, 17]. Although neomycin targets the prokaryotic 30S ribosomal subunit and causes codon misreading and mistranslation, it has been used in eukaryotes because of the similarity between mitochondrial and chloroplast ribosomes and bacterial ribosomes [18]. Zeocin intercalates in the DNA of both bacteria and eukaryotes and introduces double-stranded breaks, ultimately causing cell death [19].

## Results

To establish appropriate selection compounds for use with *Bd* and *Bsal*, we first identified antibiotics commonly used for selection with both mammalian and fungal systems. We chose five compounds (Hygromycin, Blasticidin, Puromycin, Zeocin, and Neomycin) to test based on the mechanism of action of each compound, their proven efficacy for use with both animal and fungal cells, and the availability of resistance genes (**Table 1**). We next tested the ability of these five compounds to inhibit the growth of *Bd* and *Bsal* cells in liquid culture. Although solid agar media is typically used for colony selection in chytrid and other fungi [8, 20, 21], we chose to use liquid culture to identify initial working concentrations because measuring zoospore release in liquid media is rapid and easily quantified.

**Table 1:** Antibiotic concentrations used to select for gene expression in select eukaryotes. This table lists the key features of the antibiotics used in this study: the drug class, the target, known resistance genes, the current listed price per gram from Millipore Sigma, and the concentrations used in select eukaryotes. Species include representatives from plants (*Arabidopsis thaliana* and *Chlamydomonas reinhardtii*), protozoa (*Trypanosoma brucei*), amoebae (*Dictyostelium discoideum*), fungi (*Aspergillus spp*., *Schizosaccharomyces pombe*, *Saccharomyces cerevisiae*), and animals (human) in addition to the two species tested in this study. The lowest concentrations of each antibiotic which inhibited growth in liquid and solid media for *Bd* and *Bsal* are listed from our findings in this study. These concentrations were used to calculate the cost per liter of growth media for both *Bd* and *Bsal*.

	Drug Class	Target	Known Resistance Genes	List price per gram (MilliporeSigma)	Lowest drug conc. for growth inhibition for *Bd*	Cost per Liter for *Bd*	Lowest drug conc. for growth inhibition for *Bsal*	Cost per Liter for *Bsal*	Conc. for HeLa cells	Conc. for hESC	Conc. for Fibroblasts	Conc. for *Arabidopsis thaliana*	Conc. for *Dictyostelium discoideum*	Conc. for *Trypanosoma brucei*	Conc. for *Chlamydomonas reinhardtii*	Conc. for *Aspergillus spp*	Conc. for *S*. *pombe*	Conc. for *S*. *cerevisiae*
**Neomycin**	Amino-glycoside	Ribosome [18]	*neo*‡	$1.93/g	Liquid: 600 μg/ml Solid: 1 mg/ml	Liquid: $1.16/L Solid: $1.93/L	Liquid: 250 μg/ml Solid: N/A	Liquid: $0.48/L Solid: >$1.93/L	-	-	-	-	-	-	300 μg/ml [22]	200–400 mg/ml [23]	0.375 g/L [24]	6.25 mM [25]
**Hygromycin**	Atypical Amino-glycoside	Ribosome [15]	*hyg*, *hph*	$998/g	Liquid: 1 μg/ml Solid: 0.1 μg/ml	Liquid: $1.00/L Solid: $0.10/L	Liquid: 10 μg/ml Solid: 10 μg/ml	Liquid: $9.98/L Solid: $9.98/L	100–200 μg/ml [26,27]	40 μg/ml [28]	40 μg/ml [29]	15–50 μg/ml [30,31]	25–40 μg/ml [32]	5–50 μg/ml [33,34]	1–20 μg/ml [35]	100 μg/ml [36]	400 mg/L [37]	300 μg/ml [38]
**Blasticidin**	Nucleoside Antibiotic	Ribosome [16]	*bsr*, *bls*, *bsd*	$6280/g	Liquid: 5 μg/ml Solid: 10 μg/ml	Liquid: $31.25/L Solid:$62.80	Liquid: 2 μg/ml Solid: 10 μg/ml	Liquid: $12.56/L Solid: $62.80/L	10–20 μg/ml [39,40]	2.0 μg/ml [41]	8 μg/ml [29]	10 μg/ml [42]	10 μg/ml [43,44]	2–10 μg/ml [45–47]	-	-	30 μg/ml [48,49]	10 mg/ml [50]
**Puromycin**	Amino-nucleoside	Ribosome [17]	*pac*	$5340/g	Liquid: N/A Solid: 100 μg/ml	Liquid: >$1068/L Solid: $534/L	Liquid: 50 μg/ml Solid: N/A	Liquid: $267/L Solid: >$2670/L	1–2 μg/ml [51–53]	0.5–5 μg/ml [41,54,55]	2 μg/ml [29]	-	-	0.1 μg/ml [56]	-	-	-	*200 uM [57]
**Zeocin**	Glyco-peptide Antibiotic	DNA [19]	*ble*	$177/g (Invivogen)	Liquid: 10 μg/ml Solid: 10 μg/ml	Liquid: $1.77/L Solid: $1.77/L	Liquid: 1 μg/ml Solid: 10 μg/ml	Liquid: $0.18/L Solid: $1.77/L	50 μg/ml [58]	300 μg/ml [59]	800 μg/ml [29]	100 μg/ml [60]	100 mg/L [61]	-	5–15 μg/ml [62,63]	100–125 μg/ml [64]	150 mg/ml [65]	-

- no references were found.

* the organism had to be made susceptible for the antibiotic to work.

‡ the *neo* resistance gene is also used for resistance to the drug G418 which was not tested in this study.

**Fig 2:**
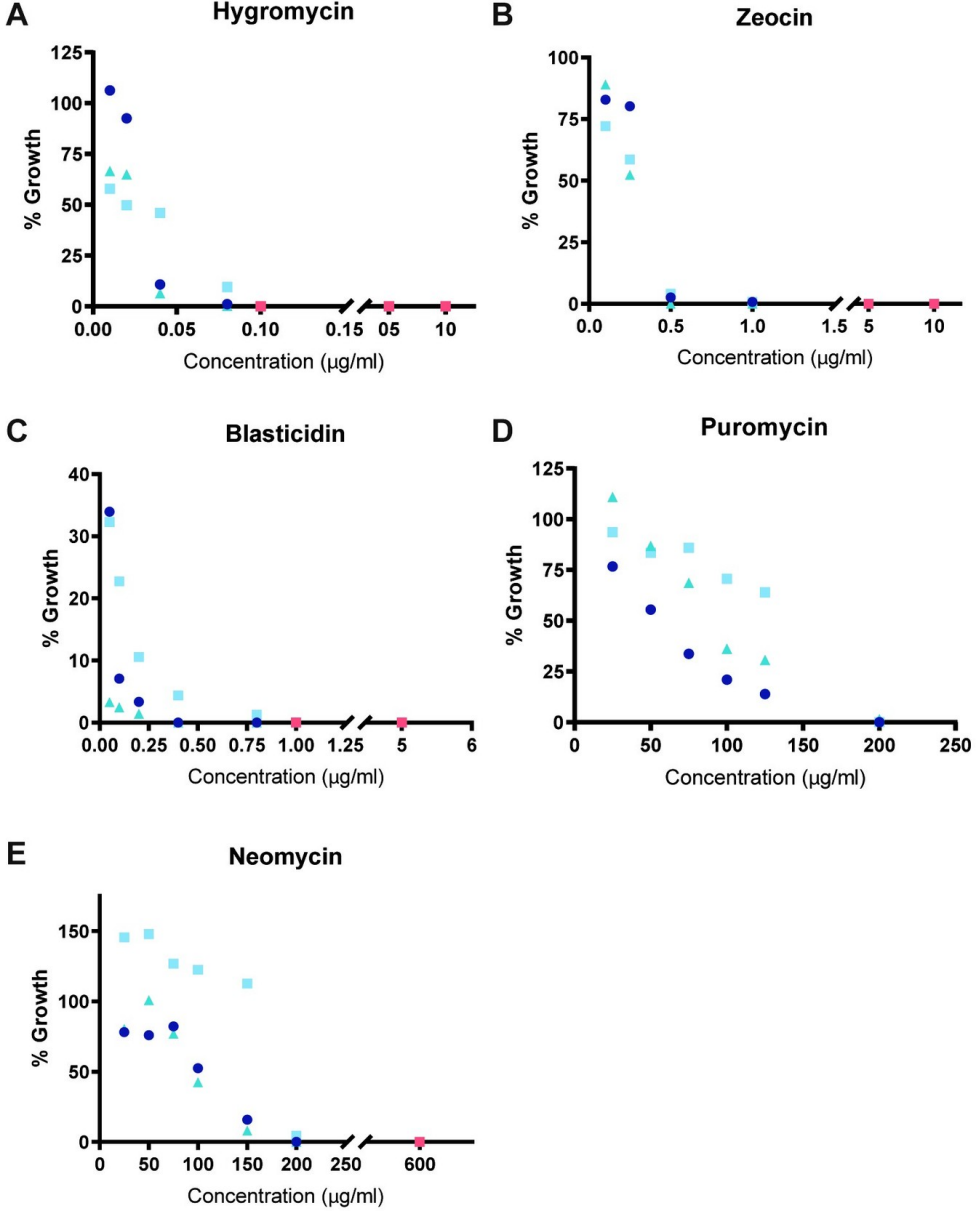
Inhibition of Bd growth in liquid media. Percent of *Bd* growth in liquid media supplemented with (A) Hygromycin, (B), Zeocin, (C) Blasticidin, (D) Puromycin, and (E) Neomycin as compared to an antibiotic free control for three temporally isolated replicates (circle, square, and triangle, shades of blue). Orange symbols indicate concentrations at which no growth occurred after three days in all three replicates.

**Fig 3:**
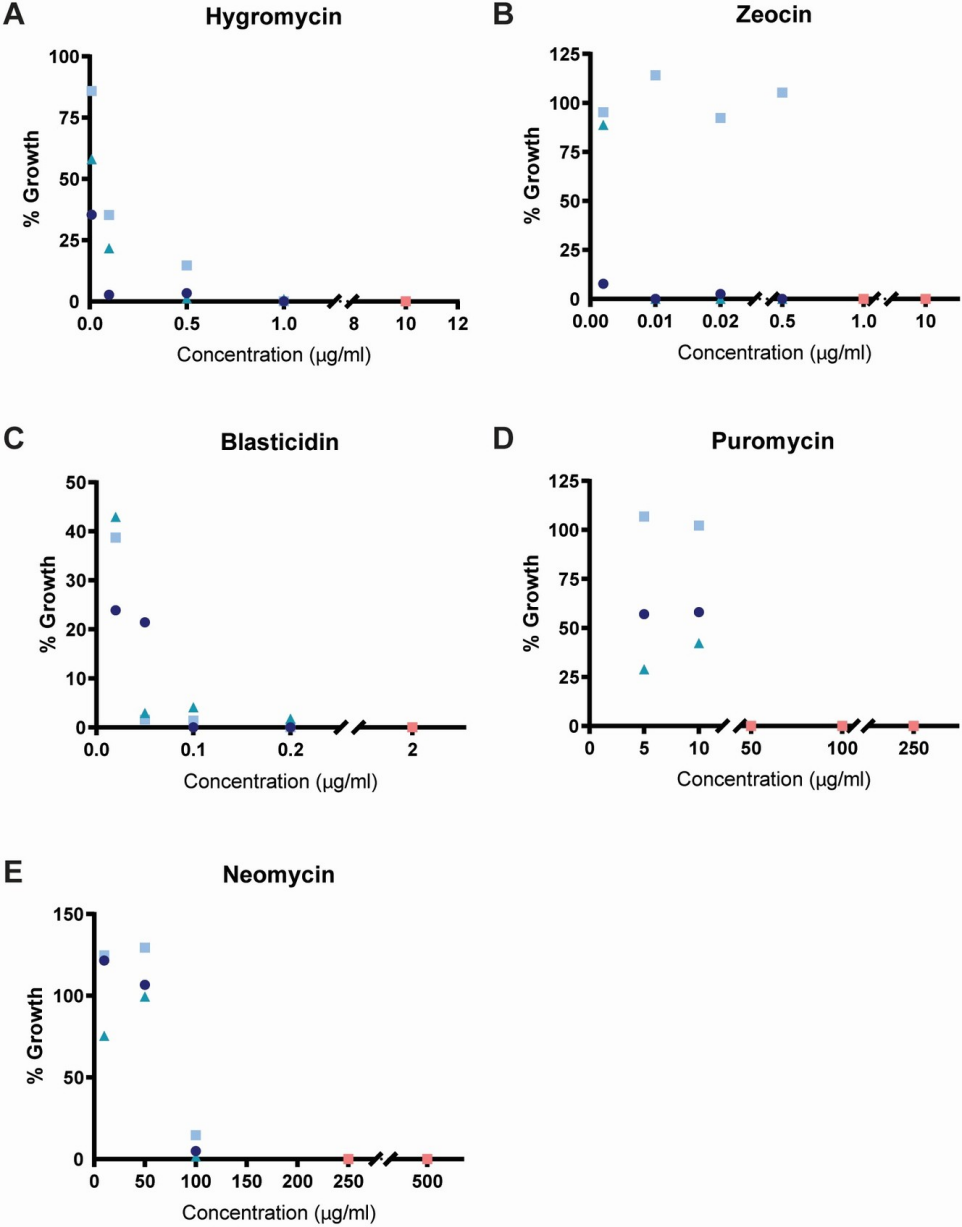
Inhibition of *Bsal* growth in liquid media. Percent of *Bsal* growth in liquid media supplemented with (A) Hygromycin, (B), Zeocin, (C) Blasticidin, (D) Puromycin, and (E) Neomycin as compared to an antibiotic free control for three temporally isolated replicates (circle, square, and triangle, shades of blue). Orange symbols indicate concentrations at which no growth occurred after four days in all three replicates.

To measure the effect of each antibiotic on *Bd* and *Bsal* growth, we added a wide range of antibiotic concentrations to cultures of age matched zoospores and allowed them to grow for one full life cycle: three (*Bd*) or four (*Bsal*) days. We then measured the concentration of released zoospores in each culture. Initial concentrations were selected based on known inhibitory concentrations for other organisms (**Table 1**) and spanned many orders of magnitude. Based on these preliminary experiments (not shown), we then identified possible working concentration ranges for each antibiotic in both species and tested intermediate concentrations using three biological replicates separated in time (**Figs 2 and 3**). To enable comparison of zoospore release from replicate experiments conducted on different days, we normalized counts for each replicate to its antibiotic-free control.

We identified antibiotic concentrations that consistently prevented growth in all three biological replicates—the successful concentrations are highlighted in orange in each figure. We found Hygromycin, Zeocin, Blasticidin and Neomycin could inhibit *Bd* growth in liquid culture (**Fig 2**), while all of the tested antibiotics inhibited *Bsal* growth (**Fig 3**). In *Bd*, Hygromycin has the lowest minimum inhibitory concentration (0.1 μg/ml), followed by Blasticidin (1 μg/ml), Zeocin (5 μg/ml), and Neomycin (600 μg/ml). Puromycin did not inhibit growth in *Bd* with the concentrations tested. In *Bsal*, Zeocin prevented growth at 1 μg/ml, followed by Blasticidin (2 μg/ml), Hygromycin (10 μg/ml), Puromycin (50 μg/ml), and Neomycin (250 μg/ml).

**Fig 4:**
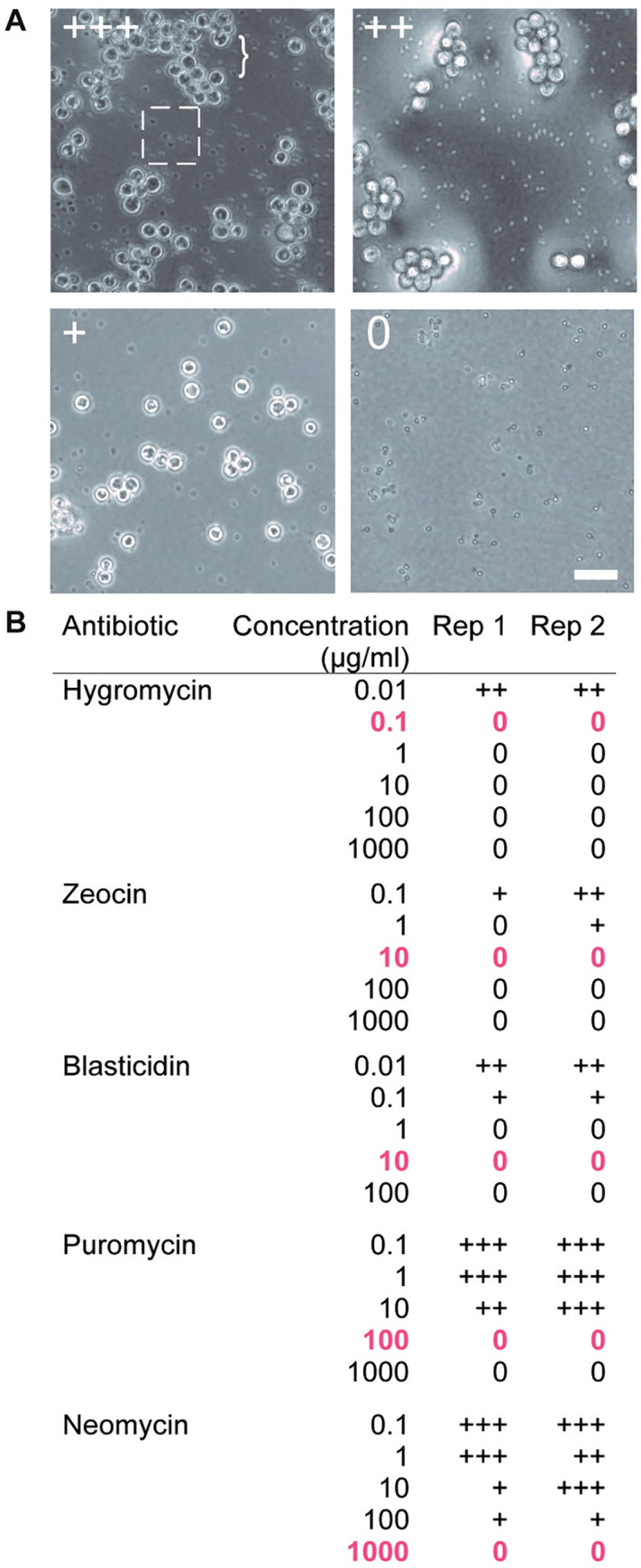
Inhibition of Bd growth on solid media. (**A**) Examples of *Bd* growth after three days on antibiotic selection plates. The ‘+’ demonstrates the relative zoospore activity of each plate compared to an antibiotic-free control plate. The box highlights zoospores, which appear as small dots while the bracket highlights sporangia. The zoospores in the ‘0’ image are immotile (see S1 Video). Scale bar 50 μm. (**B**) *Bd* growth on antibiotic selection plates. Concentrations highlighted in bold and orange are the lowest concentrations that prevent growth for at least 14 days post zoospore plating.

**Fig 5:**
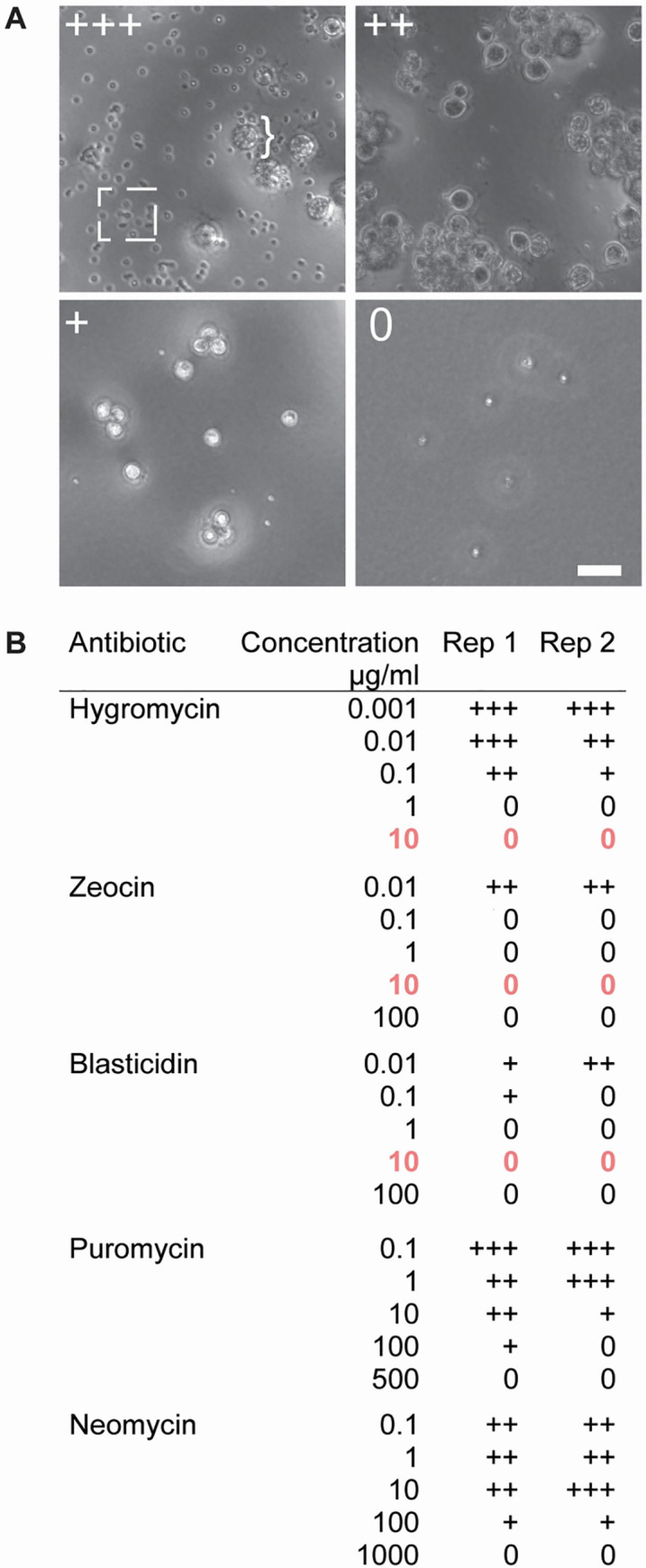
Inhibition of *Bsal* growth on solid media. (**A**) Examples of *Bsal* growth after four days on antibiotic selection plates. The ‘+’ demonstrates the relative zoospore activity of each plate compared to a no antibiotic control plate. The box highlights zoospores, which appear as small dots while the bracket highlights sporangia. The zoospores in the ‘0’ image are immotile (see S1 Video). Scale bar 50 μm. (**B**) *Bsal* growth on antibiotic selection plates. Concentrations highlighted in bold and orange are the lowest concentrations that prevent growth for at least 14 days post zoospore plating.

Having identified working concentrations of these compounds for use with liquid media, we next tested their efficacy on solid media. Growing cells on solid media allows for colony formation, which is useful for isolating successful and independent genetic transformants by “picking” colonies that grow under selection. To identify useful concentrations for selection on solid media, we inoculated zoospores on nutrient agar plates containing varying antibiotic concentrations. After a full growth cycle on selective media (three days for *Bd*, four days for *Bsal*), we compared zoospore release to antibiotic-free control cultures by flooding plates with water and looking for motile zoospores (**S1 and S2 Videos**). We defined successful concentrations as those which yielded no zoospore release in either replicate. We found at least one concentration for each antibiotic that prevented zoospore release in the timeframe of a typical growth cycle (**Figs 4 and 5**).

Because detection of colony formation often requires multiple growth cycles, we evaluated the efficiency of growth inhibition by growing plates with no zoospore release for 14 days. We found that all the tested antibiotics inhibited *Bd* growth on solid media, but only Hygromycin, Blasticidin and Zeocin inhibited growth in *Bsal*. For *Bd*, Hygromycin has the lowest minimum concentration at 0.1 μg/ml, with Blasticidin and Zeocin both following at 10 μg/ml, Puromycin at 100 μg/ml, and Neomycin at 1 mg/ml (**Fig 4**). In *Bsal*, Hygromycin, Blasticidin, and Zeocin all prevented growth for at least 14 days at a concentration of 10 μg/ml, while Puromycin and Neomycin did not prevent growth on solid media (**Fig 5**). The recommended concentrations for selection are highlighted in orange on the tables in both figures (**Figs 4B and 5B**).

## Discussion

This study identified drug concentrations that reproducibly inhibited *Bd* and *Bsal* growth in either liquid culture or on solid media. When a drug worked in both liquid culture and solid media, the solid media typically required a higher concentration of antibiotic. This may be because of the additional minerals found in the agar not present in the liquid media [66]. Hygromycin, Zeocin, and Blasticidin worked well for both species and at concentrations within the typical range used for genetic selection in other species (**Table 1**). Puromycin and Neomycin were both able to inhibit growth of *Bd* and *Bsal*, but required higher concentrations than are used for animal cell lines. Although Hygromycin, Zeocin, and Blasticidin are all effective for preventing growth of *Bd* and *Bsal*, we recommend first using Hygromycin for genetic selection because it has been successfully used for selection of transformants in the nonpathogenic chytrid *Spizellomyces punctatus*, and is widely used for other fungal species [8, 36–38].

The ability to select for genetically transformed cells will allow for tractable genetic models to facilitate hypothesis testing in *Bd* and *Bsal*. The identification of useful selection agents and appropriate working concentrations is an important first step in developing genetic tools for use with *Bd* and *Bsal*. The natural step forward will be the design of selection cassettes, most commonly in the form of transformation plasmids. We look forward to the development of these and related molecular tools that will help us answer questions about the basic cell biology of chytrids, fungal evolution, and amphibian pathology.

## Methods

### Cell growth and synchronization

*Batrachochytrium dendrobatidis* (*Bd*) isolate JEL 423 was grown in 1% (w/v) tryptone (Apex Cat. 20–251) in tissue culture treated flasks (Cell Treat 229340) at 24°C for three days. *B*. *salamandrivorans* (*Bsal*) isolate AMFP 1 was grown in half-strength TGhL liquid media (0.8% Tryptone, 0.2% gelatin hydrolysate, 0.1% lactose (w/v) in tissue culture treated flasks at 15°C for four days [67]. For both species, we synchronized the release of motile zoospores by gently washing the flask three times with fresh growth media and then incubating with 10 mL of media for 2 hours. Age matched zoospores were then collected by centrifugation at 2000 rcf for 5 mins, resuspended in media, counted, and used for experiments as outlined below.

### Drug treatments and quantitation for cells grown in liquid media

Neomycin (Fisher Cat. AAJ67011AE), Hygromycin B (Fisher Cat. AAJ60681MC), Blasticidin (Fisher Cat. BP2647100), Puromycin (Fisher Cat. BP2956100), and Zeocin (Fisher Cat. AAJ671408EQ), were screened for growth inhibition of *Bd* and *Bsal*. Cells were diluted to a starting concentration of 5x10^5^ cells/mL and 250 uL of cells were added to each well of a sterile tissue culture treated 24-well plate (Cell Treat 229123). 250 μl of appropriately diluted antibiotics and matched carrier controls were added to each well and mixed thoroughly. Plates were sealed with parafilm and grown at either 24°C for three days (*Bd*), or 15°C for four days (*Bsal*). For each of three biological replicates spaced in time, the concentration of released zoospores was estimated using the average of two independent hemocytometer counts. Zoospore concentrations were normalized to the no drug control and data plotted using Prism (GraphPad v8).

### Drug treatments and quantitation for cells grown on solid media

We added 1% agar to 50 mL batches of 1% tryptone (w/v) and half-strength TGhL then autoclaved. Each antibiotic was added to a separate, pre-cooled, 50 mL batch of media, and 10 mL of the solution added to one of five 15 mm^2^ plates (VWR 25384–090) and allowed to solidify. Equal volume of appropriate carrier liquid was added to the pre-cooled 50 mL batch of agar-media to create control plates. Plates were wrapped in parafilm and aluminum foil, and stored at 4°C. Plates were inoculated by evenly spreading 5.0 x 10^6^ zoospores across the agar and incubated at 24°C for three days (*Bd)* or 15°C for four days (*Bsal*). Three control plates were used per replicate to ensure a point of comparison if one were to be contaminated. Zoospore release was evaluated by imaging each plate for 20 seconds at one second intervals using a Nikon Ti2-E inverted microscope equipped with 10x PlanApo objective and sCMOS 4mp camera (PCO Panda) using white LED transmitted light. Approximate zoospore activity was assessed as: 0 (no visible zoospores), + (< 25% zoospore activity of control plates lacking antibiotic), ++ (~50% zoospore activity of control plates), or +++ (equivalent zoospore activity to control plates). To determine the lowest antibiotic concentration that could completely inhibit growth, plates that yielded “0” growth were allowed to grow for 14 days at the appropriate incubation temperature and reassessed as above.

## Supporting information

S1 Video *Bsal* zoospores with zero growth. Zoospores grown on antibiotic selection plates are labeled “0” if no zoospores are released or zoospores showed no growth and are immotile.
(MP4)

S2 Video *Bsal* zoospores with “+++” growth. Zoospores grown on antibiotic selection plates are labeled “+++” if the zoospore release is comparable to the no antibiotic control.
(MP4)

## References

[1] Grossart H-P, Wurzbacher C, James TY, Kagami M. Discovery of dark matter fungi in aquatic ecosystems demands a reappraisal of the phylogeny and ecology of zoosporic fungi. Fungal Ecol. 2016 2;19:28–38.

[2] Kagami M, Miki T, Takimoto G. Mycoloop: chytrids in aquatic food webs. Front Microbiol [Internet]. 2014 4 22 [cited 2020 Apr 24];5 Available from: http://journal.frontiersin.org/article/10.3389/fmicb.2014.00166/abstract 10.3389/fmicb.2014.00166 PMC4001071 24795703

[3] Gleason FH, Küpper FC, Amon JP, Picard K, Gachon CMM, Marano AV, et al Zoosporic true fungi in marine ecosystems: a review. Mar Freshw Res. 2011 5 19;62(4):383–93. 10.1071/MF10282 PMC3272469 22319023

[4] Longcore JE, Pessier AP, Nichols DK. *Batrachochytrium dendrobatidis* gen. et sp. nov., a chytrid pathogenic to amphibians. Mycologia. 1999 3;91(2):219–27.

[5] Berger L, Hyatt A, Speare R, Longcore J. Life cycle stages of the amphibian chytrid Batrachochytrium dendrobatidis. Dis Aquat Organ. 2005;68:51–63. 10.3354/dao068051 16465834

[6] Martel A, Spitzen-van der Sluijs A, Blooi M, Bert W, Ducatelle R, Fisher MC, et al Batrachochytrium salamandrivorans sp. nov. causes lethal chytridiomycosis in amphibians. Proc Natl Acad Sci. 2013 9 17;110(38):15325–9. 10.1073/pnas.1307356110 24003137 PMC3780879

[7] Fritz-Laylin LK, Lord SJ, Mullins RD. WASP and SCAR are evolutionarily conserved in actin-filled pseudopod-based motility. J Cell Biol. 2017 6 5;216(6):1673–88. 10.1083/jcb.201701074 28473602 PMC5461030

[8] Medina EM, Robinson KA, Bellingham-Johnstun K, Ianiri G, Laplante C, Fritz-Laylin LK, et al Genetic transformation of Spizellomyces punctatus, a resource for studying chytrid biology and evolutionary cell biology. Rokas A, Baldwin IT, Stearns T, editors. eLife. 2020 5 11;9:e52741 10.7554/eLife.52741 32392127 PMC7213984

[9] Bellard C, Genovesi P, Jeschke JM. Global patterns in threats to vertebrates by biological invasions. Proc R Soc B Biol Sci. 2016 1 27;283(1823):20152454. 10.1098/rspb.2015.2454 PMC4795027 26817767

[10] Olson DH, Aanensen DM, Ronnenberg KL, Powell CI, Walker SF, Bielby J, et al Mapping the Global Emergence of Batrachochytrium dendrobatidis, the Amphibian Chytrid Fungus. Stajich JE, editor. PLoS ONE. 2013 2 27;8(2):e56802 10.1371/journal.pone.0056802 23463502 PMC3584086

[11] Scheele BC, Pasmans F, Skerratt LF, Berger L, Martel A, Beukema W, et al Amphibian fungal panzootic causes catastrophic and ongoing loss of biodiversity. Science. 2019 3 29;363(6434):1459–63. 10.1126/science.aav0379 30923224

[12] Swafford AJM, Hussey SP, Fritz-Laylin LK. High-efficiency electroporation of chytrid fungi. Sci Rep. 2020 12;10(1):15145 10.1038/s41598-020-71618-2 32934254 PMC7493940

[13] Smith HO, Danner DB, Deich RA. Genetic Transformation. Annu Rev Biochem. 1981;50(1):41–68. 7023362 10.1146/annurev.bi.50.070181.000353

[14] Stepanenko AA, Heng HH. Transient and stable vector transfection: Pitfalls, off-target effects, artifacts. Mutat Res Mutat Res. 2017 7 1;773:91–103. 10.1016/j.mrrev.2017.05.002 28927539

[15] Borovinskaya MA, Shoji S, Fredrick K, Cate JHD. Structural basis for hygromycin B inhibition of protein biosynthesis. RNA. 2008 6 20;14(8):1590–9. 10.1261/rna.1076908 18567815 PMC2491480

[16] Svidritskiy E, Ling C, Ermolenko DN, Korostelev AA. Blasticidin S inhibits translation by trapping deformed tRNA on the ribosome. Proc Natl Acad Sci. 2013 7 23;110(30):12283–8. 10.1073/pnas.1304922110 23824292 PMC3725078

[17] Pestka S. Inhibitors of Ribosome Functions. 1971. 1971;25:487–562. 10.1146/annurev.mi.25.100171.002415 4949424

[18] Mehta R, Champney WS. Neomycin and Paromomycin Inhibit 30S Ribosomal Subunit Assembly in Staphylococcus aureus. Curr Microbiol. 2003 9 1;47(3):237–43. 10.1007/s00284-002-3945-9 14570276

[19] Chankova SG, Dimova E, Dimitrova M, Bryant PE. Induction of DNA double-strand breaks by zeocin in Chlamydomonas reinhardtii and the role of increased DNA double-strand breaks rejoining in the formation of an adaptive response. Radiat Environ Biophys. 2007 10 10;46(4):409–16. 10.1007/s00411-007-0123-2 17639449

[20] de Groot MJA, Bundock P, Hooykaas PJJ, Beijersbergen AGM. Agrobacterium tumefaciens-mediated transformation of filamentous fungi. 1998;16:4. 10.1038/nbt0998-839 9743116

[21] Bundock P, den Dulk-Ras A, Beijersbergen A, Hooykaas PJ. Trans-kingdom T-DNA transfer from Agrobacterium tumefaciens to Saccharomyces cerevisiae. EMBO J. 1995 7 3;14(13):3206–14. 7621833 10.1002/j.1460-2075.1995.tb07323.x PMC394382

[22] Hasnain SE, Manavathu EK, Leung WC. DNA-mediated transformation of Chlamydomonas reinhardi cells: use of aminoglycoside 3’-phosphotransferase as a selectable marker. Mol Cell Biol. 1985 12;5(12):3647–50. 10.1128/mcb.5.12.3647 3018525 PMC369200

[23] Dong Y, Cui C-B, Li C-W, Hua W, Wu C-J, Zhu T-J, et al Activation of Dormant Secondary Metabolite Production by Introducing Neomycin Resistance into the Deep-Sea Fungus, Aspergillus versicolor ZBY-3. Mar Drugs. 2014 Jul 29;12(8):4326–52. 10.3390/md12084326 PMC4145319 25076061

[24] Bureik M, Bruck N, Hubel K, Bernhardt R. The human mineralocorticoid receptor only partially differentiates between different ligands after expression in fission yeast. FEMS Yeast Res. 2005 4;5(6–7):627–33. 10.1016/j.femsyr.2004.12.007 15780662

[25] Shimma Y-I, Uno I. Isolation and characterization of neomycin-sensitive mutants in Saccharomyces cerevisiae. J Gen Microbiol. 1990;136:1753–61.

[26] Jiang H, Su ZZ, Lin JJ, Goldstein NI, Young CS, Fisher PB. The melanoma differentiation associated gene mda-7 suppresses cancer cell growth. Proc Natl Acad Sci. 1996 8 20;93(17):9160–5. 10.1073/pnas.93.17.9160 8799171 PMC38612

[27] Buchschacher GL, Panganiban AT. Human Immunodeficiency Virus Vectors for Inducible Expression of Foreign Genest. J VIROL. 1992;66:9. 10.1128/jvi.66.5.2731-2739.1992 PMC241028 1560523

[28] Sakurai K, Shimoji M, Tahimic CGT, Aiba K, Kawase E, Hasegawa K, et al Efficient integration of transgenes into a defined locus in human embryonic stem cells. Nucleic Acids Res. 2010 4;38(7):e96 10.1093/nar/gkp1234 20071742 PMC2853137

[29] Sato M, Ohtsuka M, Miura H, Miyoshi K, Watanabe S. Determination of the Optimal Concentration of Several Selective Drugs Useful for Generating Multi-Transgenic Porcine Embryonic Fibroblasts. Reprod Domest Anim. 2012;47(5):759–65. 10.1111/j.1439-0531.2011.01964.x 22136322

[30] Harrison SJ, Mott EK, Parsley K, Aspinall S, Gray JC, Cottage A. A rapid and robust method of identifying transformed Arabidopsis thaliana seedlings following floral dip transformation. Plant Methods. 2006;2(1):19. 17087829 10.1186/1746-4811-2-19 PMC1636043

[31] Rashid A. Comparison of a kanamycin versus hygromycin resistance gene in transgenic plant selection of Arabidopsis thaliana L. J Cell Sci Mutat [Internet]. 2017 [cited 2020 Apr 24];01(01). Available from: http://www.alliedacademies.org/articles/comparison-of-a-kanamycin-versus-hygromycin-resistance-gene-in-transgenic-plant-selection-of-arabidopsis-thaliana-l.html

[32] Egelhoff TT, Brown SS, Manstein DJ, Spudich JA. Hygromycin resistance as a selectable marker in Dictyostelium discoideum. Mol Cell Biol. 1989 5;9(5):1965–8. 10.1128/mcb.9.5.1965 2546056 PMC362988

[33] Biebinger S, Elizabeth Wirtz L, Lorenz P, Christine Clayton. Vectors for inducible expression of toxic gene products in bloodstream and procyclic Trypanosoma brucei. Mol Biochem Parasitol. 1997 3;85(1):99–112. 10.1016/s0166-6851(96)02815-0 9108552

[34] Wirtz E, Leal S, Ochatt C, Cross GeorgeAM. A tightly regulated inducible expression system for conditional gene knock-outs and dominant-negative genetics in Trypanosoma brucei. Mol Biochem Parasitol. 1999 3;99(1):89–101. 10.1016/s0166-6851(99)00002-x 10215027

[35] Berthold P, Schmitt R, Mages W. An Engineered Streptomyces hygroscopicus aph 7″ Gene Mediates Dominant Resistance against Hygromycin B in Chlamydomonas reinhardtii. Protist. 2002 12;153(4):401–12. 10.1078/14344610260450136 12627869

[36] Cullen D, Leong SA, Wilson LJ, Henner DJ. Transformation of Aspergillus nidulans with the hygromycin-resistance gene, hph. Gene. 1987 1;57(1):21–6. 10.1016/0378-1119(87)90172-7 3322945

[37] Brown S, Lorenz A. Single-step Marker Switching in Schizosaccharomyces pombe Using a Lithium Acetate Transformation Protocol. BIO-Protoc [Internet]. 2016 [cited 2020 Jan 23];6(24). Available from: https://bio-protocol.org/e2075 10.21769/BioProtoc.2075 PMC5366253 28352647

[38] Goldstein AL, McCusker JH. Three new dominant drug resistance cassettes for gene disruption in Saccharomyces cerevisiae. Yeast. 1999;15(14):1541–53. 10.1002/(SICI)1097-0061(199910)15:14&lt;1541::AID-YEA476&gt;3.0.CO;2-K 10514571

[39] Jin Q, Marsh J, Cornetta K, Alkhatib G. Resistance to human immunodeficiency virus type 1 (HIV-1) generated by lentivirus vector-mediated delivery of the CCR5Δ32 gene despite detectable expression of the HIV-1 co-receptors. J Gen Virol. 2008 10;89(Pt 10):2611–21. 10.1099/vir.0.2008/003624-0 18796731 PMC2679520

[40] Cheng N, He R, Tian J, Ye PP, Ye RD. Cutting Edge: TLR2 Is a Functional Receptor for Acute-Phase Serum Amyloid A. J Immunol. 2008 7 1;181(1):22–6. 10.4049/jimmunol.181.1.22 18566366 PMC2464454

[41] Moore JC, Atze K, Yeung PL, Toro-Ramos AJ, Camarillo C, Thompson K, et al Efficient, high-throughput transfection of human embryonic stem cells. Stem Cell Res Ther. 2010 7 26;1(3):23 10.1186/scrt23 20659329 PMC2941115

[42] Tamura K, Kimura M, Yamaguchi I. Blasticidin S Deaminase Gene (BSD): a new selection marker gene for transformation of Arabidopsis thaliana and Nicotiana tabacum. Biosci Biotech Biochem. 1995;59(12):2336–8. 10.1271/bbb.59.2336 8611760

[43] Thompson CRL, Kay RR. The Role of DIF-1 Signaling in Dictyostelium Development. Mol Cell. 2000 12;6(6):1509–14. 10.1016/s1097-2765(00)00147-7 11163223

[44] Li G, Alexander H, Schneider N, Alexander S. Molecular basis for resistance to the anticancer drug cisplatin in Dictyostelium. 2019;9. 10.1099/00221287-146-9-2219 10974109

[45] Brooks DR, McCulloch R, Coombs GH, Mottram JC. Stable transformation of trypanosomatids through targeted chromosomal integration of the selectable marker gene encoding blasticidin S deaminase. FEMS Microbiol Lett. 2000 5;186(2):287–91. 10.1111/j.1574-6968.2000.tb09119.x 10802186

[46] Roper JR, Güther MLS, MacRae JI, Prescott AR, Hallyburton I, Acosta-Serrano A, et al The Suppression of Galactose Metabolism in Procylic Form *Trypanosoma brucei* Causes Cessation of Cell Growth and Alters Procyclin Glycoprotein Structure and Copy Number. J Biol Chem. 2005 5 20;280(20):19728–36. 10.1074/jbc.M502370200 15767252

[47] Erben ED, Fadda A, Lueong S, Hoheisel JD, Clayton C. A Genome-Wide Tethering Screen Reveals Novel Potential Post-Transcriptional Regulators in Trypanosoma brucei. Tschudi C, editor. Pathog PLoS. 2014 Jun 12;10(6):e1004178. 10.1371/journal.ppat.1004178 PMC4055773 24945722

[48] Zhang X-R, He J-B, Wang Y-Z, Du L-L. A Cloning-Free Method for CRISPR/Cas9-Mediated Genome Editing in Fission Yeast. G3amp58 GenesGenomesGenetics. 2018 6;8(6):2067–77. 10.1534/g3.118.200164 PMC5982833 29703785

[49] Kimura M, Kamakura T, Zhou Tao Q, Kaneko I, Yamaguchi I. Cloning of the blasticidin S deaminase gene (BSD) from Aspergillus terreus and its use as a selectable marker for Schizosaccharomyces pombe and Pyricularia oryzae. Mol Gen Genet MGG. 1994 1 1;242(2):121–9. 10.1007/BF00391004 8159161

[50] Fukuda H, Kizaki Y. A new transformation system of Saccharomyces cerevisiae with blasticidin S deaminase gene. 1999;3.

[51] Ukekawa R, Miki K, Fujii M, Hirano H, Ayusawa D. Accumulation of multiple forms of lamin A with down-regulation of FACE-1 suppresses growth in senescent human cells. Genes Cells. 2007 3;12(3):397–406. 10.1111/j.1365-2443.2007.01057.x 17352743

[52] Huang J, Dibble CC, Matsuzaki M, Manning BD. The TSC1-TSC2 Complex Is Required for Proper Activation of mTOR Complex 2. Mol Cell Biol. 2008 6 15;28(12):4104–15. 10.1128/MCB.00289-08 18411301 PMC2423120

[53] Grueneberg DA, Degot S, Pearlberg J, Li W, Davies JE, Baldwin A, et al Kinase requirements in human cells: I. Comparing kinase requirements across various cell types. Proc Natl Acad Sci. 2008 10 28;105(43):16472–7. 10.1073/pnas.0808019105 18948591 PMC2575444

[54] Yang C, Przyborski S, Cooke MJ, Zhang X, Stewart R, Anyfantis G, et al A Key Role for Telomerase Reverse Transcriptase Unit in Modulating Human Embryonic Stem Cell Proliferation, Cell Cycle Dynamics, and In Vitro Differentiation. STEM CELLS. 2008;26(4):850–63. 10.1634/stemcells.2007-0677 18203676

[55] Drobinskaya I, Linn T, Šarić T, Bretzel RG, Bohlen H, Hescheler J, et al Scalable Selection of Hepatocyte- and Hepatocyte Precursor-Like Cells from Culture of Differentiating Transgenically Modified Murine Embryonic Stem Cells. STEM CELLS. 2008;26(9):2245–56. 10.1634/stemcells.2008-0387 18556507

[56] Niemirowicz GT, Cazzulo JJ, Álvarez VE, Bouvier LA. Simplified inducible system for Trypanosoma brucei. PLoS ONE [Internet]. 2018 10 11 [cited 2020 Apr 24];13(10). Available from: https://www.ncbi.nlm.nih.gov/pmc/articles/PMC6181392/10.1371/journal.pone.0205527 PMC6181392 30308039

[57] Cary GA, Yoon SH, Torres CG, Wang K, Hays M, Ludlow C, et al Identification and characterization of a drug-sensitive strain enables puromycin-based translational assays in Saccharomyces cerevisiae. Yeast. 2014;31(5):167–78. 10.1002/yea.3007 24610064 PMC4013229

[58] Mesnil M, Piccoli C, Tiraby G, Willecke K, Yamasaki H. Bystander killing of cancer cells by herpes simplex virus thymidine kinase gene is mediated by connexins. Proc Natl Acad Sci. 1996 3 5;93(5):1831–5. 10.1073/pnas.93.5.1831 8700844 PMC39867

[59] Chow MZY, Geng L, Kong C-W, Keung W, Fung JC-K, Boheler KR, et al Epigenetic Regulation of the Electrophysiological Phenotype of Human Embryonic Stem Cell-Derived Ventricular Cardiomyocytes: Insights for Driven Maturation and Hypertrophic Growth. Stem Cells Dev. 2013 10;22(19):2678–90. 10.1089/scd.2013.0125 23656529 PMC3780424

[60] Van Hove J, Fouquaert E, Smith DF, Proost P, Van Damme EJM. Lectin activity of the nucleocytoplasmic EUL protein from Arabidopsis thaliana. Biochem Biophys Res Commun. 2011 10;414(1):101–5. 10.1016/j.bbrc.2011.09.031 21945438 PMC3195871

[61] Nicolussi A, Dunn JD, Mlynek G, Bellei M, Zamocky M, Battistuzzi G, et al Secreted heme peroxidase from *Dictyostelium discoideum*: Insights into catalysis, structure, and biological role. J Biol Chem. 2018 1 26;293(4):1330–45. 10.1074/jbc.RA117.000463 29242189 PMC5787809

[62] Liu P, Lechtreck KF. The Bardet–Biedl syndrome protein complex is an adapter expanding the cargo range of intraflagellar transport trains for ciliary export. Proc Natl Acad Sci. 2018 1 30;115(5):E934–43. 10.1073/pnas.1713226115 29339469 PMC5798339

[63] López‐Paz C, Liu D, Geng S, Umen JG. Identification of *Chlamydomonas reinhardtii* endogenous genic flanking sequences for improved transgene expression. Plant J. 2017 12;92(6):1232–44. 10.1111/tpj.13731 28980350 PMC5718938

[64] Han G, Shao Q, Li C, Zhao K, Jiang L, Fan J, et al An efficient Agrobacterium-mediated transformation method for aflatoxin generation fungus Aspergillus flavus. J Microbiol. 2018 5;56(5):356–64. 10.1007/s12275-018-7349-3 29721833

[65] Benko Z, Zhao RY. Zeocin for selection of bleMX6 resistance in fission yeast. BioTechniques. 2011 7 1;51(1):57–60. 10.2144/000113706 21781055

[66] Tam JP, Lu Y-A, Yang J-L. Correlations of Cationic Charges with Salt Sensitivity and Microbial Specificity of Cystine-stabilized β-Strand Antimicrobial Peptides. J Biol Chem. 2002 12 27;277(52):50450–6. 10.1074/jbc.M208429200 12399464

[67] Robinson KA, Pereira KE, Bletz MC, Carter ED, Gray MJ, Piovia-Scott J, et al Isolation and maintenance of Batrachochytrium salamandrivorans cultures. Dis Aquat Organ. 2020 6 18;140:1–11. 10.3354/dao03488 32618283

